# Comprehensive Phenotypic Characterization of Clinical *Elizabethkingia* Isolates and Evaluation of the Antimicrobial and Anti-Biofilm Activity of Dialdehyde Cellulose

**DOI:** 10.3390/ijms27146392

**Published:** 2026-07-18

**Authors:** Sastra Yuantrakul, Orathai Yinsai, Tanpong Chaiwarit, Punnaporn Srisithan, Wenting Zhou, Kritsana Ruenjai, Phadungkiat Khamnoi, Kwanjit Duangsonk

**Affiliations:** 1 Department of Microbiology, Faculty of Medicine, Chiang Mai University, Chiang Mai 50200, Thailand; medxchamp@gmail.com (S.Y.); orathai.yinsai@gmail.com (O.Y.); punnaporn.ps@gmail.com (P.S.); wenting_z@cmu.ac.th (W.Z.); 2 Office of Research Administration, Chiang Mai University, Chiang Mai 50200, Thailand; 3 Department of Pharmaceutical Sciences, Faculty of Pharmacy, Chiang Mai University, Chiang Mai 50200, Thailand; tanpong.ch@cmu.ac.th (T.C.); kritsana_r@cmu.ac.th (K.R.); 4 Guangxi Technology Innovation Cooperation Base of Prevention and Control Pathogenic Microbes with Drug Resistance, Youjiang Medical University for Nationalities, Baise 533000, China; 5 Microbiology Section, Diagnostic Laboratory, Maharaj Nakorn Chiang Mai Hospital, Chiang Mai 50200, Thailand; phadungkiat.k@cmu.ac.th

**Keywords:** *Elizabethkingia*, antimicrobial resistance, dialdehyde cellulose, biofilm formation, multidrug-resistant, nosocomial pathogen, virulence factors, anti-biofilm activity, carbapenem resistance, infection control

## Abstract

*Elizabethkingia* species have emerged as important nosocomial pathogens associated with multidrug resistance and persistent infections. This study aimed to characterize clinical *Elizabethkingia* isolates from Northern Thailand regarding antimicrobial susceptibility, virulence-associated phenotypes, and biofilm formation, and to evaluate the antimicrobial and anti-biofilm activity of dialdehyde cellulose (DAC) film. A total of 49 clinical isolates were identified by MALDI-TOF mass spectrometry, with species identification confirmed by 16S rRNA gene sequencing. Antimicrobial susceptibility was determined against 12 agents. Virulence traits (protease, lipase, lecithinase, and hemolysin production) and biofilm formation were assessed using standard phenotypic assays. DAC films were evaluated against selected resistant isolates. *Elizabethkingia anophelis* predominated, and most isolates exhibited multidrug or extensive drug resistance, with high resistance to carbapenems and cephalosporins. Piperacillin–tazobactam, levofloxacin, and trimethoprim–sulfamethoxazole showed the greatest activity. All isolates demonstrated protease production and time-dependent hemolysis, while lipase and lecithinase activities were absent. Biofilm formation varied among isolates, while DAC films inhibited bacterial growth and prevented detectable biofilm formation in the tested isolates. No significant difference was observed between DAC and DAC supplemented with meropenem in inhibition zone diameters (*p* = 0.555). Clinical *Elizabethkingia* isolates demonstrated extensive antimicrobial resistance with conserved virulence traits and heterogeneous biofilm formation. DAC films demonstrated antimicrobial activity and prevented detectable biofilm formation under the experimental conditions. Further studies are warranted to evaluate their mechanism of action and potential applications.

## 1. Introduction

*Elizabethkingia* species are non-fermentative, Gram-negative bacilli [1] belonging to the family Weeksellaceae [2], and are increasingly recognized as opportunistic pathogens in immunocompromised and critically ill patients [3,4,5]. Infections are associated with substantial morbidity and mortality [6,7], prolonged hospitalization, and clinical manifestations, including bacteremia, pneumonia, meningitis, and device-associated infections [8]. The species most frequently implicated in human disease are *Elizabethkingia anophelis* (*E. anophelis*), *Elizabethkingia meningoseptica* (*E. meningoseptica*), and *Elizabethkingia miricola* (*E. miricola*) [9], which pose particular challenges in healthcare settings [10]. Management of *Elizabethkingia* infections is complicated by intrinsic resistance to multiple antimicrobial classes, including β-lactams, carbapenems, and aminoglycosides, largely mediated by chromosomally encoded β-lactamases and additional resistance mechanisms [11,12,13]. This multidrug resistance limits therapeutic options, delays appropriate therapy, and contributes to poor clinical outcomes [14]. In addition, the ability of *Elizabethkingia* spp. to persist on medical devices and in hospital environments may further hinder infection control and facilitate transmission, potentially through biofilm-associated survival mechanisms [15].

Virulence traits, including virulence enzyme production, contribute to tissue damage and immune evasion [16], while biofilm formation enhances environmental persistence and antimicrobial tolerance, promoting chronic colonization [17,18]. Importantly, the distribution of *Elizabethkingia* species, antimicrobial resistance patterns, and virulence characteristics may vary geographically [19,20,21]. Therefore, regional investigations, including antimicrobial susceptibility profiles and phenotypic data, are critical [22] for understanding the characteristics of the local population, guiding antimicrobial stewardship, informing infection control strategies, and improving clinical outcomes [23,24,25]. Since biofilm-associated persistence remains a challenge for infection control, interest has expanded toward materials that can prevent microbial attachment in addition to exerting antimicrobial effects [26,27]. Biopolymers have gained increasing attention in medical applications because of their biocompatibility, biodegradability, and potential for functional modification [28,29]. Among these materials, dialdehyde cellulose (DAC), an oxidized cellulose derivative, has emerged as a promising antimicrobial biomaterial [30]. Previous studies demonstrated that DAC possesses antibacterial activity against both Gram-positive and Gram-negative bacteria [30] and may reduce bacterial adhesion and interfere with biofilm formation [31], supporting its potential application as an antimicrobial coating for medical devices to limit microbial persistence [32]. Despite the increasing clinical importance of *Elizabethkingia* species, the antimicrobial activity of dialdehyde cellulose (DAC) against these multidrug-resistant pathogens has not been investigated. Therefore, evaluating DAC against *Elizabethkingia* may provide insight into its potential as an antimicrobial biomaterial for controlling bacterial growth and biofilm formation.

This study aimed to comprehensively characterize the epidemiology, demographics, species distribution, and clinical sources of *Elizabethkingia* isolates collected from patients at Maharaj Nakorn Chiang Mai Hospital, as well as to investigate their antimicrobial susceptibility profiles and phenotypic traits, including virulence enzyme production and biofilm formation capacity. Furthermore, this study aimed to evaluate the antimicrobial properties of DAC and assess its antibiofilm efficacy against four distinct biofilm phenotypes exhibited by selected *Elizabethkingia* isolates.

## 2. Results

### 2.1. Characteristics of Elizabethkingia Isolates and Species Distribution

A total of 49 non-duplicate *Elizabethkingia* isolates were collected from 49 clinical specimens obtained from patients at Maharaj Nakorn Chiang Mai Hospital. The majority of isolates were from sputum, accounting for 78% of cases. Additional sources included bronchoalveolar lavage (*n* = 2, 4%), hemoculture (*n* = 4, 8%), tip of catheter (*n* = 1, 2%), contact lens 1 (2%), urine 2 (4%), and bile 1 (2%). Species identification by 16S rRNA sequencing revealed three *Elizabethkingia* species: *E. anophelis* (*n* = 41, 83.67%), *E. meningoseptica* (*n* = 6, 12.25%), and *E. miricola* (*n* = 2, 4.08%). Isolate codes were assigned according to species designation: CMEA for *E. anophelis*, CMEM for *E. meningoseptica*, and CMEMi for *E. miricola*. All isolates were derived from hospitalized patients, except for one contact lens isolate obtained from an outpatient at the Eye Clinic. The predominance of respiratory specimens is consistent with the recognized association of *Elizabethkingia* spp. with respiratory tract colonization and infection.

### 2.2. Antimicrobial Susceptibility Profiles

The isolates were tested for antimicrobial susceptibility against 12 antimicrobial agents. Overall antimicrobial susceptibility results are summarized in Table 1, while species-specific susceptibility distributions are shown in Table 2. Detailed isolate-level MIC values and interpretations are provided in Supplementary Table S1. Of 49 isolates, 38 (77.55%) isolates were classified as multidrug-resistant (MDR), 9 (18.37%) as extensively drug-resistant (XDR), and only 2 (4.08%) as susceptible. Resistance was pronounced among carbapenems and most cephalosporins. Ceftazidime showed the highest resistance rate (97.96%), followed by imipenem and meropenem, exhibiting resistance rates of 85.72% and 89.8%, respectively, and MIC_50_/MIC_90_ values uniformly in the resistant range (>16 µg/mL). This indicates widespread resistant to carbapenems and cephalosporins in this population. The susceptibility rates among the antimicrobial agents tested ranged from 0% to 91.84%. Piperacillin-tazobactam exhibited highest susceptibility rates (91.84%), followed by levofloxacin (83.67%) and trimethoprim-sulfamethoxazole (81.63%), respectively, which make them agents exhibiting the highest in vitro susceptibility, confirmed by their MIC_50_ values falling within susceptible range (≤8, 1, 2 µg/mL) despite high MIC_90_ values (>8 µg/mL and >4 µg/mL) of levofloxacin and trimethoprim-sulfamethoxazole, only MIC_90_ for piperacillin-tazobactam falls in susceptible value (16 µg/mL) make them cover most population in this study and most susceptible agent. Across the three species, piperacillin-tazobactam, levofloxacin, and trimethoprim-sulfamethoxazole remained the most effective agents, with *E. anophelis* exhibiting a more resistant profile overall. Additionally, ciprofloxacin demonstrated high susceptibility rates for *E. meningoseptica* (83.33%) and *E. miricola* (100%).

### 2.3. Phenotypic Characteristics: Enzyme Production, Hemolysis, and Biofilm Formation

All 49 isolates were screened for selected virulence-associated phenotypes, including protease, lipase, lecithinase, and hemolysin. Qualitative analysis of enzyme production is indicated in Table 2, with detailed data provided in Supplementary Table S2. Protease activity was commonly detected among *Elizabethkingia* isolates. At 24 h, 43 isolates (87.76%) demonstrated detectable protease production, and all isolates were positive by 48 h. Among the species, *E. anophelis* showed 90.24% positivity at 24 h, while *E. miricola* exhibited 100% positivity at the same time point. In contrast, *E. meningoseptica* demonstrated a relatively lower positivity rate (66.66%) at 24 h, suggesting comparatively delayed protease production in this species during early incubation. However, lipase and lecithinase activities were uniformly absent across all *Elizabethkingia* isolates at every time point (24, 48, and 72 h). Representative images of enzyme production and hemolysis are provided in Supplementary Figures S1 and S2.

Protease activity was assessed by the presence of a clear zone around bacterial growth and was recorded at 24, 48, and 72 h. Lipase and lecithinase activities were determined by the formation of an opaque precipitate and an iridescent sheen, respectively, at 72 h of incubation. Data are presented as the number and percentage of positive isolates for each species and in total.

For the hemolysis test, all 49 strains demonstrated alpha-hemolytic activity with the pattern evolving over time. At 24 h, alpha-hemolysis was predominant, observed in 44 isolates (89.8%), while 5 isolates (10%) showed no detectable hemolysis. By 48 h, 46 isolates (93.88%) exhibited alpha-hemolysis, and 3 (6.12%) exhibited beta-hemolysis. At 72 h, beta-hemolysis became the dominant phenotype, observed in 45 isolates (91.84%), with 4 isolates (8.16%) showing alpha-hemolysis.

The biofilm-forming capacity results for the 49 *Elizabethkingia* isolates are shown in Table 3. Overall, biofilm formation varied widely across isolates, ranging from non-producers to strong biofilm formers. More than half of the isolates in this study were classified as biofilm producers, with the majority exhibiting a strong phenotype (18/29), indicating a high ability to adhere to surfaces. *E. anophelis* demonstrated the highest heterogeneity in biofilm formation among the three species. Within *E. meningoseptica*, five out of six isolates were strong biofilm producers, while one was a non-biofilm former; notably, moderate and weak phenotypes were absent in this species. In contrast, all *E. miricola* isolates were classified as non-biofilm formers under the tested conditions. Representative images of the biofilm formation assay are provided in Supplementary Figure S3.

### 2.4. Antimicrobial Activity of Dialdehyde Cellulose (DAC) Film Against Elizabethkingia anophelis

#### 2.4.1. Phenotypic Validation of Tested Isolates and Controls

Antimicrobial activity of DAC was evaluated and is shown in Table 4 and Supplementary Figure S5. The inhibition zone diameters produced by the control antibiotic disks including meropenem and TMP/SMX (trimethoprim-sulfamethoxazole) were consistent with the previously characterized antimicrobial susceptibility profiles of the tested isolates (Supplementary Table S2). All four *E. anophelis* isolates exhibited susceptibility to TMP/SMX, with inhibition zones ranging from 18.50 ± 0.87 to 24.33 ± 0.58 mm, whereas no inhibition zone was observed with meropenem, confirming their meropenem-resistant phenotype. In contrast, the reference strains *E. coli* and *P. aeruginosa* demonstrated large inhibition zones in response to meropenem (34.67 ± 0.76 mm and 32.67 ± 0.58 mm, respectively). *E. coli* was also susceptible to TMP/SMX, exhibiting an inhibition zone of 30.83 ± 0.76 mm.

#### 2.4.2. Antimicrobial Efficacy of DAC Against *E. anophelis* and Controls

DAC films exhibited antimicrobial activity against all tested *E. anophelis* isolates, producing inhibition zones ranging from 22.33 ± 0.58 to 26.00 ± 2.08 mm. Similarly, DAC combined with meropenem (DAC + meropenem) produced inhibition zones ranging from 23.17 ± 0.29 to 25.00 ± 1.00 mm. Notably, the antimicrobial activity of DAC was observed in all *E. anophelis* isolates despite the absence of detectable activity of meropenem alone. The inhibition zones generated by DAC and DAC + meropenem were comparable to those produced by TMP/SMX across all tested isolates. Furthermore, in isolates CMEA36 and CMEA89, the inhibition zones produced by DAC and DAC + meropenem were numerically larger than those produced by TMP/SMX. (Supplementary Figure S6) Antimicrobial activity was consistently observed among all four clinical isolates, with relatively low variability between replicate measurements.

#### 2.4.3. Statistical Evaluation of DAC-Meropenem Interaction

Comparison with the reference strains revealed a markedly different activity profile. Against *P. aeruginosa*, DAC and DAC + meropenem produced inhibition zones of 9.67 ± 0.58 mm and 9.00 ± 0.00 mm, respectively, compared with 32.67 ± 0.58 mm for meropenem. Similarly, against *E. coli*, DAC and DAC + meropenem generated inhibition zones of 9.33 ± 0.58 mm and 11.50 ± 0.50 mm, respectively, which were substantially smaller than those produced by TMP/SMX and meropenem. Overall, inhibition zones were numerically larger for the tested *E. anophelis* isolates than for the reference strains (*E. coli* and *P. aeruginosa*); however, this difference did not reach statistical significance (Mann–Whitney U test, *p* = 0.1333). No significant difference was observed between DAC and DAC + meropenem inhibition zone diameters across the tested organisms (paired *t*-test, *p* = 0.555), indicating that incorporation of meropenem did not measurably enhance the antimicrobial activity of DAC under the experimental conditions evaluated.

### 2.5. Preliminary Evaluation of the Biofilm Inhibitory Effect of DAC-Coated Plates

The capacity of DAC film in biofilm formation inhibition in 4 selected *Elizabethkingia* isolates was evaluated using the crystal violet assay. Four *E. anophelis* clinical isolates (CMEA29, CMEA36, CMEA89, CMEA112) representing distinct baseline biofilm phenotypes (moderate-, weak-, non-, and strong-biofilm producers, respectively) were selected for evaluation.

As summarized in Table 5, all tested isolates showed visible growth after 24 h under control conditions, and the observed biofilm phenotypes were concordant with the results obtained from the crystal violet assay (Supplementary Table S2), confirming the reproducibility of the experimental model. Exposure to DAC-coated films resulted in growth inhibition of all four selected *E. anophelis* isolates, independent of their intrinsic biofilm-forming phenotype. In contrast, the growth of *E. coli* and *P. aeruginosa* remained detectable at the 24 h time point. (Supplementary Figure S4).

Notably, despite the growth of control strains, no detectable biofilm formation was observed in the control strains cultured on DAC-coated plates. The introduction of DAC films inhibited biofilm formation of all tested and control strains. These findings provide preliminary evidence that DAC inhibits biofilm formation, as assessed by the crystal violet assay, across organisms with diverse biofilm-forming capacities. Because bacterial growth was completely inhibited in the tested *E. anophelis* isolates, this study cannot determine whether the observed reduction in biofilm formation resulted from a direct anti-biofilm effect or was secondary to growth inhibition.

## 3. Discussion

This study characterized antimicrobial susceptibility, virulence-associated phenotypes, and the antimicrobial and preliminary biofilm inhibitory potential of DAC among clinical *Elizabethkingia* isolates collected in Northern Thailand. Our findings support the previous finding that clinical *Elizabethkingia* isolates exhibit extensive antimicrobial resistance [13] alongside phenotypes associated with persistence and pathogenicity [33], while DAC represents a promising alternative strategy for controlling bacterial growth and biofilm formation. Consistent with recent epidemiological reports, *E. anophelis* was the predominant species [34] identified in this study. This observation supports the growing recognition of *E. anophelis* as the major clinically relevant species within the genus [35] and highlights the importance of molecular identification for accurate surveillance and clinical interpretation [5]. The predominance of respiratory specimens further supports the association of *Elizabethkingia* with respiratory colonization and infection in hospitalized patients [36,37].

The antimicrobial susceptibility profiling revealed extensive resistance across the isolate collection, with the majority of isolates classified as MDR or XDR and resistance concentrated predominantly against carbapenems and cephalosporins. These findings align with previous studies describing intrinsic resistance mechanisms in *Elizabethkingia*, particularly reduced susceptibility to β-lactam agents [38,39]. A large retrospective study of *E. meningoseptica* clinical isolates (*n* = 208) similarly reported high resistance rates to carbapenems and cephalosporins, with piperacillin-tazobactam and trimethoprim-sulfamethoxazole demonstrating the most favorable susceptibility profiles, consistent with our observations [40]. The high level of carbapenem resistance observed emphasizes concerns regarding the limited effectiveness of empirical carbapenem therapy against this organism [41]. By contrast, piperacillin–tazobactam, levofloxacin, and trimethoprim–sulfamethoxazole retained the highest activity among the agents tested. However, elevated MIC_90_ values for several agents indicate considerable susceptibility heterogeneity within the isolate population, emphasizing the importance of isolate-specific susceptibility testing to guide therapeutic decision-making [42].

Beyond antimicrobial resistance, *Elizabethkingia* isolates demonstrated phenotypes associated with pathogenic persistence and host invasion [43,44]. Protease activity was highly prevalent, becoming detectable in all isolates following prolonged incubation, whereas lipase and lecithinase activities were not detected in any isolates under the experimental conditions employed. Previous studies have similarly reported proteolytic activity and identified protease-associated virulence determinants in *Elizabethkingia* [39], supporting the role of extracellular proteases in nutrient acquisition, host interaction, and adaptation to host environments [45,46]. The consistently high prevalence of protease production observed in the present study further supports proteolytic activity as an important virulence-associated characteristic among clinical *Elizabethkingia* isolates. A previous study reported positive lecithinase activity and detectable extracellular lipase activity in *E. meningoseptica* [47]. In contrast, neither lecithinase nor lipase activity was detected in any *Elizabethkingia* isolate in the present study, including *E. meningoseptica*, under the experimental conditions tested. This discrepancy suggests that expression of these extracellular enzymes may not be constitutive and could instead vary according to strain background, culture conditions, assay methodology, or environmental factors [48,49,50]. The hemolytic pattern observed in this study was consistent with previous findings [51]. Most isolates (46/49) exhibited α-hemolysis after 48 h of incubation before transitioning to β-hemolysis at 72 h. This temporal shift suggests that hemolytic phenotype may be time-dependent, highlighting the importance of standardized observation conditions when comparing virulence-associated characteristics across studies. Notably, integration of these virulence-associated phenotypes did not allow classification of *E. anophelis* isolates into high- and low-virulence groups, as protease activity and β-hemolysis were observed in all isolates, while lipase and lecithinase activities were uniformly absent. These phenotypes therefore appear to reflect conserved, species-associated traits rather than markers predictive of clinical severity or treatment outcome. Taken together with the observed variability in biofilm formation, these findings suggest that the persistence of *Elizabethkingia* in clinical settings likely results from the combined effects of antimicrobial resistance and regulated virulence-associated adaptation, rather than resistance alone [52,53].

Biofilm formation was commonly observed but varied substantially among isolates. A previous study by Hu et al. (2022) reported that all *E. anophelis* isolates produced strong biofilms [54]; in contrast, this study found that nearly half of the isolates (40.82%) were non-biofilm producers. No significant association was observed between specimen type and biofilm phenotype patterns, with the exception of bloodstream isolates, which predominantly exhibited non-biofilm formation, with only one isolate identified as a weak biofilm producer, consistent with previous findings [54].

As previously described, biofilm formation may contribute to increased antimicrobial resistance [55,56,57]. However, no relationship was observed between biofilm formation and susceptibility to the antimicrobial agents evaluated in this study (piperacillin–tazobactam, levofloxacin, and trimethoprim–sulfamethoxazole). Consistent with previous findings [58,59], no significant correlation was observed between resistance profiles (susceptible, XDR, and MDR) and biofilm-forming phenotype. Instead, the relationship between resistance and biofilm formation appears to be strain- and agent-dependent, varying across studies according to the antimicrobials and the isolates tested [54,58,59].

Since biofilm development contributes to antimicrobial tolerance, environmental survival, and chronic infection [60,61], these findings further support the importance of surface-associated persistence in *Elizabethkingia* pathogenesis and highlight biofilm formation as a potential therapeutic target [62,63]. Another major finding of this study was the demonstrated antimicrobial activity of DAC against four meropenem-resistant *E. anophelis* isolates, selected to represent four different biofilm phenotypes, including non-, weak, moderate-, and strong-biofilm producers (all protease-positive and lipase- and lecithinase-negative). This selection enabled the investigation of the association between biofilm phenotype and the effects of DAC on growth inhibition and biofilm reduction. DAC demonstrated antimicrobial activity against all four tested *Elizabethkingia* isolates and the two reference strains. The inhibition zones observed with DAC were of a similar numerical magnitude to those produced by trimethoprim–sulfamethoxazole. These findings support previous observations that carboxylated dialdehyde cellulose (CDAC) film exhibits antimicrobial activity against *E. coli* and *P. aeruginosa* using a comparable experimental approach [64]. Incorporation of meropenem did not demonstrate a significant synergistic effect, despite previous studies suggesting that CDAC may exhibit synergy with clindamycin [64]. One possible explanation is that meropenem activity was partially reduced during film preparation or sterilization [65], as β-lactam antibiotics are known to be susceptible to degradation under suboptimal processing conditions [66]. Alternatively, the antimicrobial activity observed may have primarily originated from DAC itself, potentially masking any additional contribution from incorporated meropenem. As antibiotic stability, loading efficiency, and release kinetics were not evaluated in this study, these explanations remain hypothetical and require further investigation. Although the precise antimicrobial mechanism of DAC was not directly examined, oxidized cellulose-derived materials have been proposed to act through mechanisms distinct from those of conventional antibiotics, including disruption of bacterial surface interactions and interference with cellular integrity [67]. Prior research indicates that higher aldehyde content in DAC-based materials enhances antibacterial activity. This effect is attributed to the reaction of aldehyde groups with surface amines, compromising structure and leading to bacterial inactivation [68,69]. The activity observed against meropenem-resistant isolates therefore suggests that DAC may retain efficacy independently of established antibiotic resistance mechanisms.

CDAC was previously proposed as a promising material for treating wound infections on viable surfaces [64]. Extending this concept, our findings suggest that DAC may also inhibit bacterial growth and biofilm formation on inanimate surfaces, indicating broader antimicrobial and preliminary biofilm-inhibitory potential beyond living tissue applications. Notably, DAC prevented detectable biofilm formation across *E. anophelis* isolates representing different intrinsic biofilm phenotypes. In the tested *E. anophelis* isolates, biofilm inhibition on DAC-coated surfaces coincided with complete growth suppression, and an independent anti-biofilm mechanism cannot be confirmed for these isolates from the present data [70]. DAC inhibited bacterial growth and prevented detectable biofilm formation under the tested conditions; whether DAC possesses a growth-independent anti-biofilm mechanism remains to be determined. The observation of biofilm inhibition despite persistent growth in the reference strains raises this possibility but requires confirmation through further mechanistic study, such as parallel quantification of viable cell counts (CFU/mL) alongside biofilm biomass [71]. This observation broadens the potential application of DAC beyond conventional antimicrobial treatment and supports its possible role as a surface-modifying strategy for preventing microbial persistence [72].

From a translational perspective, the preliminary biofilm inhibitory effects of DAC-coated surfaces highlight their potential as a medical coating platform. Biofilm-associated colonization remains a major challenge for medical devices, as attached bacterial communities resist antimicrobials and drive persistent infections [73]. Materials capable of reducing colonization and preventing biofilm establishment thus offer a valuable preventive strategy [74,75]. Given the increasing prominence of *Elizabethkingia* spp. as persistent healthcare-associated pathogens, targeting early adhesion and biofilm formation may offer advantages beyond those achievable with conventional therapy alone [76]. Furthermore, the antimicrobial properties of DAC film suggest potential applications in wound dressings and barrier materials, where preventing bacterial colonization could support infection control and reduce device- or procedure-related infections [74].

Several limitations of this study should be acknowledged. First, isolates were obtained from a single center, and evaluation of DAC was limited to representative strains and phenotypic outcomes. Although 49 clinical isolates were included, only two *E. miricola* isolates were available; therefore, observations for this species should be interpreted with caution and are descriptive rather than intended for robust statistical comparison. Larger studies incorporating greater isolate diversity and additional bacterial species are needed to improve statistical robustness and strengthen generalizability. Second, the molecular mechanisms underlying antimicrobial resistance, virulence expression, and DAC-mediated antimicrobial activity were not investigated, and meropenem stability following incorporation and sterilization was not assessed. Third, only selected antimicrobial agents were evaluated; future studies should incorporate a broader antimicrobial panel to more comprehensively characterize susceptibility patterns and to determine the effects of antimicrobial incorporation into DAC films. Fourth, disc diffusion methodology, originally developed and validated for standardized small-molecule antibiotic disks [77], has recognized limitations even among conventional agents, as inhibition zone size is influenced by drug solubility, diffusion rate, and disk or film thickness; zone diameters are directly comparable only between agents with similar diffusion characteristics. As DAC is a polymeric film rather than a diffusible small molecule, direct comparison of its inhibition zones with antibiotic controls (TMP/SMX, meropenem) should be interpreted with appropriate caution, and future studies should incorporate dedicated diffusion-rate validation specific to the DAC matrix. Although additional characterization of DAC was outside the scope of the present study, further investigation of coating stability, antibiotic release kinetics, biocompatibility, SEM surface morphology, and mechanical testing under experimental conditions represents an important direction for future work.

## 4. Materials and Methods

### 4.1. Microbial Sampling, Identification, and Culture Techniques

This study included a diverse collection of *Elizabethkingia* spp. isolates from individual patients collected by the Diagnostic Laboratory of Maharaj Nakorn Chiang Mai Hospital, Thailand (2021–2025). Only non-duplicate isolates, with one isolate per patient, were included in the study. Contaminants and non-dominant isolates were excluded from the analysis.

Species identification of all bacterial isolates was initially performed using a MALDI Biotyper^®^ system (Bruker Daltonics, Billerica, MA, USA) at the diagnostic laboratory of Maharaj Nakorn Chiang Mai Hospital. To ensure accurate species-level identification, all isolates were subsequently subjected to 16S rRNA gene sequencing using the universal primer pair 27F (5′-AGAGTTTGATCMTGGCTCAG-3′) and 1492R (5′-TACGGYTACCTTGTTACGACTT-3′). The GenBank accession numbers of the 16S rRNA gene sequences analyzed in this study are provided in Supplementary Table S3. Sequence similarity searches were performed using the NCBI BLASTn web server (Basic Local Alignment Search Tool; National Center for Biotechnology Information, https://blast.ncbi.nlm.nih.gov/Blast.cgi?PROGRAM=blastn&PAGE_TYPE=BlastSearch&LINK_LOC=blasthome; accessed on 19 September 2025). Species-level identification was based on concordant results from MALDI-TOF mass spectrometry and 16S rRNA gene sequencing, with ≥99% sequence identity together with query coverage and alignment quality, thereby providing complementary proteomic and genotypic confirmation of species identity [78,79]. Isolates identified as non-*Elizabethkingia* by 16S rRNA gene sequencing or those showing <99% sequence identity were excluded from further analysis. Isolates were cultured overnight at 37 °C on Trypticase Soy Agar (TSA) (Becton, Dickinson and Company, Sparks, MD, USA). For long-term storage, all isolates were preserved at −80 °C in Trypticase Soy Broth (TSB) supplemented with 20% glycerol.

### 4.2. Culture Preparation for the Assessment of Virulence Phenotypes

To prepare bacterial cultures for the analysis of hemolysis and virulence enzyme production, glycerol stocks were streaked onto nutrient agar and incubated at 37 °C for 24 h. Specifically, two distinct colonies were carefully picked and restreaked onto a series of specialized solid culture media, each formulated to detect a specific virulence enzyme, such as lecithinase, lipase, or protease. This subculturing process was essential to observe and characterize the phenotypic expression of these pathogenicity-related traits on the respective indicator media.

### 4.3. Qualitative Virulence Enzymes Production

A distinct bacterial colony was streaked onto substrate-specific media to assess hemolysis type and the production of specific virulence enzymes. All plates were incubated at 37 °C for 72 h, a temperature suitable for the growth of all strains in this study. A qualitative analysis (scoring for presence or absence) of the reactions was then performed.

Hemolytic activity was assayed according to Buxton [80] using pre-poured 5% sheep blood agar (Sheep Blood Agar O1200, Biomedia, Bangkok, Thailand).

The production of virulence enzymes was conducted based on the method of Edberg et al. [81] with the following modifications: Protease production was tested on Tryptic Soy Agar (TSA) (Becton, Dickinson and Company, Sparks, MD, USA) supplemented with 3% (*w*/*v*) skim milk powder (TM574, Titan Biotech LTD., New Delhi, India). Lipase and lecithinase production were tested on egg yolk agar base (TM724, Titan Biotech LTD., India) supplemented with 30% (*v*/*v*) egg yolk emulsion according to the instructions (TS002, Titan Biotech LTD., India). Each enzyme activity assay was performed on three independently inoculated agar plates for each isolate.

### 4.4. Biofilm Formation and Biofilm Inhibition Assay

The biofilm-forming capacity of *Elizabethkingia* spp. isolates and the inhibitory effect of DAC were evaluated using a modified crystal violet assay adapted for *Elizabethkingia* species [54,82]. *Escherichia coli* ATCC 25922 and *Pseudomonas aeruginosa* ATCC 27853 were used as standard control strains. All experiments were performed in triplicate. Bacteria cultured for 24 h on Mueller-Hinton Agar (MHA; Oxoid, Basingstoke, UK) at 37 °C were harvested and suspended in Mueller-Hinton Broth (MHB) to achieve a 0.5 McFarland standard. For the biofilm formation assay, a 20 µL aliquot of this suspension was added to 180 µL of MHB in untreated 96-well cell culture-treated polystyrene plates (Corning Incorporated, Corning, NY, USA). For the biofilm inhibition assay, 96-well plates were first pre-coated by adding 200 µL of 4% DAC solution (*w*/*v*) per well, followed by complete evaporation to form a uniform DAC coating on the well surfaces. The coated plates were sterilized with ethylene oxide prior to bacterial inoculation, which was performed identically to the biofilm formation assay. Successful sterilization was confirmed using a chemical indicator, and no microbial growth was observed in the blank control wells throughout the biofilm inhibition assay, confirming the sterility of the coated plates. After 24 h incubation at 37 °C, both sets of plates were washed three times with 200 µL of phosphate-buffered saline (PBS; 0.01 mol/L) to remove non-adherent cells. The plates were air-dried in a 60 °C hot-air oven, after which the adherent biofilms were fixed with 200 µL of methanol for 15 min and stained with 200 µL of 1% crystal violet (Beyotime, Shanghai, China) for 15 min at room temperature. Following additional washing with PBS to remove unbound dye, the biofilm-bound crystal violet was solubilized by adding 150 µL of 33% acetic acid per well and incubating for 5 min on a shaking table. In the biofilm inhibition assay (DAC-coated plates), the solubilized solution was further diluted 1:10 with 33% acetic acid before transfer to a new 96-well plate. The optical density (OD) of each well was measured at 595 nm using an Epoch2 microtiter plate reader (BioTek, Winooski, VT, USA). The background OD value, obtained from sterile medium processed through the identical fixation and staining procedure, was subtracted from all sample readings. Based on the corrected OD595 values, isolates were categorized as non-biofilm producers (OD595 ≤ ODc), weak biofilm producers (OD595 > ODc but ≤ 2 × ODc), moderate biofilm producers (OD595 > 2 × ODc but ≤ 4 × ODc), or strong biofilm producers (OD595 > 4 × ODc), where ODc represents the mean OD of the negative control. This approach enabled the simultaneous assessment of inherent biofilm formation in uncoated wells and the preliminary biofilm inhibitory effect of DAC in pre-coated wells. The biofilm assay was performed using three independent biological replicates generated from freshly prepared bacterial cultures, and the mean values were used for analysis.

### 4.5. Antimicrobial Susceptibility Testing

The susceptibility profile of *Elizabethkingia* spp. isolates to a panel of twelve antimicrobial agents was assessed by determining the minimum inhibitory concentrations (MICs) of twelve antimicrobial agents: piperacillin-tazobactam (TZ), ceftazidime (CZ), ceftriaxone (TX), cefepime (FEP), imipenem (IP), meropenem (MP), amikacin (AN), ciprofloxacin (CI), levofloxacin (LX), cefotaxime (CX), gentamicin (G), and trimethoprim/sulfamethoxazole (ST) using the Sensititre™ ARIS™ 2X system (Thermo Fisher Scientific, Waltham, MA, USA).

Antimicrobial susceptibility testing was performed according to the manufacturer’s instructions. A 0.5 McFarland suspension was prepared from pure cultures, subsequently diluted in cation-adjusted Mueller–Hinton broth (CAMHB), and inoculated into Sensititre GNID panels. The panels were then processed using the Sensititre ARIS 2X system, which automatically incubated them at 35 ± 2 °C for 16–24 h and determined minimum inhibitory concentrations (MICs) through fluorometric detection. Results were analyzed with Sensititre SWIN™ software (version 3.3) and interpreted according to CLSI criteria as susceptible (S), intermediate (I), or resistant (R) [83]. Isolates exhibiting resistance to at least one agent in three or more antimicrobial classes were defined as MDR, whereas those resistant to at least one agent in all but two or fewer antimicrobial classes were categorized as XDR [84].

### 4.6. Preparation of DAC Film

Dialdehyde cellulose (DAC) was synthesized according to a previous study by Chaiwarit et al. (2025) [85]. A 4% (*w*/*v*) DAC solution was prepared for the biofilm inhibition assay by dissolving 4 g of DAC in 100 mL of water at 80 °C under continuous stirring until completely dissolved. The method for preparing the DAC film was modified from a previous study by Chaiwarit et al. (2024) [64]. DAC powder (aldehyde content: 9.88 mmol/g) was mixed with hydroxypropyl methylcellulose (HPMC) at a ratio of 3:1. Briefly, 3 g of DAC was dissolved in 94.4 g of deionized water at 80 °C for 4 h using a hotplate magnetic stirrer at 400 rpm. Subsequently, 1 g of HPMC was completely dissolved in the DAC solution, followed by the addition of 1.6 g of glycerol as a plasticizer. The polymeric solution was then homogenized by stirring at room temperature for 30 min. Ten grams of the resulting solution were poured into a glass Petri dish (50 × 17 mm) and dried in a hot-air oven at 40 °C.

For preparation of the meropenem-containing DAC film, meropenem trihydrate (sodium content: 3.9 mmol/g; Biolab Co., Ltd., Samut Prakan, Thailand) was dissolved in the polymeric solution at room temperature at a concentration of 21.79 mg per 100 g of solution. The dried films were cut into circular discs with a diameter of 0.6 cm, each containing 10 µg of meropenem (equivalent to a standard meropenem disk), and sterilized using ethylene oxide.

### 4.7. Antimicrobial Activity of DAC Film Against Elizabethkingia Isolates

Four *Elizabethkingia* isolates (CMEA29, CMEA36, CMEA89, andCMEA112) were selected to evaluate the antimicrobial activity of the DAC film. The isolates were chosen to represent different biofilm-forming phenotypes, including moderate-, weak-, non-, and strong-biofilm producers, respectively. Antimicrobial susceptibility testing of DAC film was performed using the agar disk diffusion assay according to Abral et al. (2021) [86], with modifications.

Prior to testing, all film samples were sterilized using ethylene oxide according to the hospital’s validated ethylene oxide sterilization protocol, which includes routine biological and chemical indicator monitoring. Representative sterilized films were additionally cultured prior to antimicrobial testing, and no microbial growth was observed. The selected *Elizabethkingia* isolates, together with standard control strains *Escherichia coli* ATCC 25922 and *Pseudomonas aeruginosa* ATCC 27853, were cultured on Mueller–Hinton agar (MHA; Oxoid, UK) at 37 °C for 24 h and subsequently inoculated into Mueller–Hinton broth (Sigma-Aldrich, St. Louis, MO, USA) and incubated aerobically at 37 °C for 12 h. Bacterial suspensions were adjusted to OD_600_ = 0.1, and 100 μL of each suspension was uniformly spread onto Mueller–Hinton agar (MHA; Oxoid, UK) plates.

DAC films and meropenem-loaded DAC films (0.6 cm. diameter) were aseptically placed onto the inoculated agar surface and incubated aerobically at 37 °C for 16–18 h. Antimicrobial activity was evaluated by measuring the diameter of the zone of inhibition surrounding each film sample. A trimethoprim–sulfamethoxazole disk was included as the positive control, whereas a meropenem disk was used as the negative control based on the antimicrobial susceptibility profiles of the selected *Elizabethkingia* isolates. As no standardized interpretive criteria are available for DAC-containing films, the presence of an inhibition zone was interpreted as evidence of antimicrobial activity, and inhibition zone diameters were used only for relative comparison among the tested films. Antimicrobial susceptibility testing of the DAC films was performed in three independent biological experiments for each isolate.

### 4.8. Statistical Analysis

Statistical analyses were performed using GraphPad Prism (version 11.0.2; GraphPad Software, San Diego, CA, USA). Comparisons between two independent groups were performed using the Mann–Whitney U test, whereas paired comparisons were analyzed using the paired *t*-test. Normality of the residuals was assessed using the Shapiro–Wilk test prior to conducting the paired *t*-test. A two-sided *p*-value < 0.05 was considered statistically significant.

## Figures and Tables

**Table 1:** Overall antimicrobial susceptibility profiles of *Elizabethkingia* spp. isolates (total; *n* = 49).

Antibiotics	Total (*n* = 49)				
	S	I	R	MIC_50_	MIC_90_
Piperacillin-Tazobactam	91.84 (45/49)	4.08 (2/49)	4.08 (2/49)	≤8 (S)	16 (S)
Ceftazidime	2.04 (1/49)	0	97.96 (48/49)	>32 (R)	>32 (R)
Ceftriaxone	0	51.02 (25/49)	48.98 (24/49)	32 (I)	>32 (R)
Cefepime	14.29 (7/49)	40.82 (20/49)	44.89 (22/49)	16 (I)	>32 (R)
Imipenem	8.16 (4/49)	6.12 (3/49)	85.72 (42/49)	>16 (R)	>16 (R)
Meropenem	4.08 (2/49)	6.12 (3/49)	89.8 (44/49)	>16 (R)	>16 (R)
Amikacin	34.69 (17/49)	38.78 (19/49)	26.53 (13/49)	32 (I)	>32 (R)
Ciprofloxacin	46.93 (23/49)	24.49 (12/49)	28.58 (14/49)	2 (I)	>2 (R)
Levofloxacin	83.67 (41/49)	2.04 (1/49)	14.29 (7/49)	1 (S)	>8 (R)
Cefotaxime	2.04 (1/49)	42.86 (21/49)	55.10 (27/49)	32 (I)	>32 (R)
Gentamicin	12.25 (6/49)	2.04 (1/49)	85.71 (42/49)	>8 (R)	>8 (R)
Trimethoprim/ Sulfamethoxazole	81.63 (40/49)	0	18.37 (9/49)	2 (S)	>4 (R)

Susceptibility results for *Elizabethkingia* isolates are shown for each tested antimicrobial agent. Results are presented as percentages, with the number of isolates in parentheses. They are categorized as susceptible (S), intermediate (I), or resistant (R) based on the applied interpretive criteria. MIC_50_, A minimum concentration value at which 50% of the isolates were inhibited; MIC_90_, A minimum concentration value at which 90% of the isolates were inhibited.

**Table 2:** Protease, lipase, and lecithinase activities of *Elizabethkingia* species.

Species (No. of Isolates)	Protease (Positive)			Lipase (Positive at 72 h)	Lecithinase (Positive at 72 h)
	24 h	48 h	72 h		
*E. anophelis* (*n* = 41)	37(90.24%)	41 (100%)	41 (100%)	0 (0%)	0 (0%)
*E. meningoseptica* (*n* = 6)	4(66.66%)	6 (100%)	6 (100%)	0 (0%)	0 (0%)
*E. miricola* (*n* = 2)	2 (100%)	2 (100%)	2 (100%)	0 (0%)	0 (0%)
Total (*n* = 49)	43(87.76%)	49 (100%)	49 (100%)	0 (0%)	0 (0%)

**Table 3:** Species-specific biofilm-forming abilities of 49 *Elizabethkingia* species.

Species	Total Isolates	Strong	Moderate	Weak	Non
*E. anophelis*	41	13 (31.71%)	5 (12.20%)	6 (14.64%)	17 (41.46%)
*E. meningoseptica*	6	5 (83.33%)	0	0	1 (16.67%)
*E. miricola*	2	0	0	0	2 (100%)
Total	49	18 (36.74%)	5 (10.20%)	6 (12.26%)	20 (40.82%)

Biofilm formation was categorized as strong, moderate, weak, or non-biofilm based on optical density measurements. Data are presented as the number and percentage of isolates for each species and in total.

**Table 4:** Antimicrobial activity of DAC film and DAC combined with meropenem against clinical *E. anophelis* isolates and reference bacterial strains as determined by disk diffusion assay.

**Organism**	Inhibition Zone (mm.)	**TMP/SMX**	**Meropenem**	**Meropenem + DAC**	**DAC**
*Pseudomonas* *aeruginosa*		-	32.67 ± 0.58	9 ± 0	9.67 ± 0.58
*Escherichia coli*		30.83 ± 0.76	34.67 ± 0.76	11.50 ± 0.5	9.33 ± 0.58
CMEA29		24.33 ± 0.578	-	23.17 ± 0.29	24 ± 0
CMEA36		21 ± 1	-	25 ± 1	26 ± 2.08
CMEA89		18.50 ± 0.87	-	23.67 ± 0.76	23 ± 0.5
CMEA112		22.67 ± 0.29	-	24.17 ± 0.29	22.33 ± 0.58

Data are presented as mean inhibition zone diameter (mm) ± SD from three independent experiments. Trimethoprim–sulfamethoxazole (TMP/SMX) and meropenem were included as antimicrobial control agents. CMEA29, CMEA36, CMEA89, and CMEA112 represent clinical *E. anophelis* isolates. *Escherichia coli* and *Pseudomonas aeruginosa* were used as reference strains. “-“ indicates no detectable inhibition zone.

**Table 5:** Effect of DAC film on bacterial growth and biofilm formation.

Organism	Control		DAC Coated Plate	
	Growth	Biofilm Formation	Growth	Biofilm Formation
*Pseudomonas aeruginosa*	+	Strong	+	–
*Escherichia coli*	+	Strong	+	–
CMEA29	+	Moderate	–	–
CMEA36	+	Weak	–	–
CMEA89	+	Non	–	–
CMEA112	+	Strong	–	–

Bacterial growth and biofilm formation were evaluated after 24 h incubation in uncoated control plates and DAC-coated plates using *E. coli* and *P. aeruginosa* as reference strains and selected *E. anophelis* isolates representing non-, weak-, moderate-, and strong-biofilm phenotypes. Symbols: (+), detectable growth or biofilm; (−), no detectable growth or biofilm.

## Data Availability

The original contributions presented in this study are included in the article/Supplementary Material. Further inquiries can be directed to the corresponding author.

## Supplementary Materials

The following supporting information can be downloaded at https://www.mdpi.com/article/10.3390/ijms27146392/s1.

## References

[1] Peng S.Y., Chen L.K., Wu W.J., Paramita P., Yang P.W., Li Y.Z., Lai M.J., Chang K.C. (2020). Isolation and Characterization of a New Phage Infecting *Elizabethkingia anophelis* and Evaluation of Its Therapeutic Efficacy in vitro and in vivo. Front. Microbiol..

[2] Schoch C.L., Ciufo S., Domrachev M., Hotton C.L., Kannan S., Khovanskaya R., Leipe D., Mcveigh R., O’nEill K., Robbertse B. (2020). NCBI Taxonomy: A comprehensive update on curation, resources and tools. Database.

[3] Chang J., Kim S., Kwak Y.G., Um T.H., Cho C.R., Song J.E. (2023). Clinical and Microbiological Characteristics of Chryseobacterium indologenes Bacteremia: A 20-Year Experience in a Single University Hospital. Infect. Chemother..

[4] Baruah F.K., Borkakoty B., Ahmed A., Bora P. (2020). Neonatal Meningitis and Septicemia Caused by Multidrug-Resistant *Elizabethkingia anophelis* Identified by 16s Ribosomal RNA: An Emerging Threat. J. Glob. Infect. Dis..

[5] Tan J.W.Y., Lian B.J.X., Loh C.Y.X., See K.C. (2026). *Elizabethkingia* Species as an Emerging Pathogen: A Comprehensive Review of Clinical and Microbiological Evidence. Pathogens.

[6] Jiang B., Zhang W., Deng N., Li G., Ren C., Sun F., Wang X., Xi S., Wei S. (2025). A systematic review of reported symptomatic *Elizabethkingia* infection cases in children and adults. Acta Trop..

[7] Ngiam J.N., Koh M.C.Y., Foo T.J., Allen D.M., Chew K.L. (2025). Risk factors for mortality in *Elizabethkingia* spp. bloodstream infections. J. Glob. Antimicrob. Resist..

[8] Lau S.K., Chow W.N., Foo C.H., Curreem S.O., Lo G.C., Teng J.L., Chen J.H., Ng R.H., Wu A.K., Cheung I.Y. (2016). *Elizabethkingia anophelis* bacteremia is associated with clinically significant infections and high mortality. Sci. Rep..

[9] Dziuban E.J., Franks J.L., So M., Peacock G., Blaney D.D. (2018). *Elizabethkingia* in Children: A Comprehensive Review of Symptomatic Cases Reported From 1944 to 2017. Clin. Infect. Dis..

[10] Fahmy M., Stewart A., Tey S.K., Hajkowicz K. (2024). *Elizabethkingia* bloodstream infections in severely immunocompromised patients: Persistent, relapsing and associated with high mortality. JAC Antimicrob. Resist..

[11] DeFrank A., Bosco M., Muñoz Gómez S., Dhaliwal A. (2025). Cefiderocol-Resistant *Elizabethkingia anophelis* Bacteremia Following WATCHMAN Implantation: A Case Report and Review of the Literature. Case Rep. Infect. Dis..

[12] Hem S., Jarocki V.M., Baker D.J., Charles I.G., Drigo B., Aucote S., Donner E., Burnard D., Bauer M.J., Harris P.N.A. (2022). Genomic analysis of *Elizabethkingia* species from aquatic environments: Evidence for potential clinical transmission. Curr. Res. Microb. Sci..

[13] Jasim S.A., Mohammed J.S., Roopashree R., Alshahrani M.Y., Sharma A., Sharma A., Asliddin S., Beig M. (2025). Unraveling the global landscape of *Elizabethkingia* antibiotic resistance: A systematic review and meta-analysis. PLoS ONE.

[14] Chuang W.L., Chang F.C., Kuo C.F., Lin C.C. (2025). Multidrug-Resistant *Elizabethkingia anophelis* Bacteremia in Northern Taiwan: Focusing on Prognostic Factors and Antimicrobial Susceptibility to Minocycline and Rifampin. Infect. Drug Resist..

[15] Shen J., Weng Y., Shimada T., Karan M., Watson A., Medernach R.L., Young V.B., Hayden M.K., Hartmann E.M. (2026). Hospital Environments Harbor Chlorhexidine-Tolerant Bacteria Potentially Linked to Chlorhexidine Persistence in the Environment. Environ. Sci. Technol..

[16] Hussein Almola A., Al-Omari A.W., Younis Mahdy Al-Hamadany A. (2022). Molecular Study of Acinetobacter baumannii that Lacking Some Essentials Genes Responsible of Toxin-Antitoxin System. Arch. Razi Inst..

[17] Mishra A., Aggarwal A., Khan F. (2024). Medical Device-Associated Infections Caused by Biofilm-Forming Microbial Pathogens and Controlling Strategies. Antibiotics.

[18] Holcomb D.A., Riner D., Cowan B., Salah Z., Jennings W.C., Mattioli M.C., Murphy J.L. (2024). Chlorine Inactivation of *Elizabethkingia* spp. in Water. Emerg. Infect. Dis..

[19] Huang C. (2024). Antimicrobial Susceptibility Patterns and Antimicrobial Therapy of Infections Caused by *Elizabethkingia* Species. Medicina.

[20] Alhaj Sulaiman A., Bakleh M.Z., Ali R., Aouida M., Ramotar D. (2025). *Elizabethkingia* in the MENA region (2000–2024): A review of distribution, antimicrobial resistance, and clinical features. BMC Infect. Dis..

[21] Cai Y., Shi Q., Yu S., Li H., Yang Y., Wang D., Tung T.H., Shen B., Chen M. (2025). Epidemiological and genomic features of clinical isolates of the *Elizabethkingia* genus in Taizhou City, China. J. Glob. Antimicrob. Resist..

[22] Hayden M.K., Sansom S.E., Snitkin E.S. (2026). Genome sequencing for prevention of health-care-associated bacterial infections. Nat. Rev. Microbiol..

[23] Jauneikaite E., Baker K.S., Nunn J.G., Midega J.T., Hsu L.Y., Singh S.R., Halpin A.L., Hopkins K.L., Price J.R., Srikantiah P. (2023). Genomics for antimicrobial resistance surveillance to support infection prevention and control in health-care facilities. Lancet Microbe.

[24] Sherry N.L., Horan K.A., Ballard S.A., Gonҫalves da Silva A., Gorrie C.L., Schultz M.B., Stevens K., Valcanis M., Sait M.L., Stinear T.P. (2023). An ISO-certified genomics workflow for identification and surveillance of antimicrobial resistance. Nat. Commun..

[25] Okeke I.N., Feasey N., Parkhill J., Turner P., Limmathurotsakul D., Georgiou P., Holmes A., Peacock S.J. (2020). Leapfrogging laboratories: The promise and pitfalls of high-tech solutions for antimicrobial resistance surveillance in low-income settings. BMJ Glob. Health.

[26] Patra S., Saha S., Singh R., Tomar N., Gulati P. (2025). Biofilm battleground: Unveiling the hidden challenges, current approaches and future perspectives in combating biofilm associated bacterial infections. Microb. Pathog..

[27] Hernández-Huerta M.T., Pérez-Campos E., Pérez-Campos Mayoral L., Vásquez Martínez I.P., Reyna González W., Jarquín González E.E., Aldossary H., Alhabib I., Yamani L.Z., Elhadi N. (2025). Proactive Strategies to Prevent Biofilm-Associated Infections: From Mechanistic Insights to Clinical Translation. Microorganisms.

[28] Abdulsalam L., Abubakar S., Permatasari I., Lawal A.A., Uddin S., Ullah S., Ahmad I. (2025). Advanced Biocompatible and Biodegradable Polymers: A Review of Functionalization, Smart Systems, and Sustainable Applications. Polymers.

[29] Park J., De R. (2025). Next-Generation Wound Healing Materials: Role of Biopolymers and Their Composites. Polymers.

[30] Mayer S., Tallawi M., De Luca I., Calarco A., Reinhardt N., Gray L.A., Drechsler K., Moeini A., Germann N. (2021). Antimicrobial and physicochemical characterization of 2,3-dialdehyde cellulose-based wound dressings systems. Carbohydr. Polym..

[31] Mahdy A.S., Hassan E.A., El-Sayyad G.S., Taher H.A., Zayed S.E. (2025). Effect of gold nanoparticles and γ-cyclodextrin polymer on physicochemical, antimicrobial, and antibiofilm properties of a novel schiff base derived from dialdehyde cellulose and camphor thiazole-imine. BMC Microbiol..

[32] Athukoralalage S.S.A., Datson Z., Darwish N., Zhu Y., Chung K.H.K., Chew K., Rowan A.E., Amiralian N. (2025). Dual-Functional Antimicrobial and Low-Fouling Cellulose Coatings. ACS Appl. Mater. Interfaces.

[33] Xiong J., Han T., Hu J., Wu Y., Huang X., Yang D., Hou W., Lin Y. (2025). Genomic profiling of *Elizabethkingia anophelis* clinical isolates from a Shanghai hospital: Phylogenetic divergence coexists with heterogeneous antibiotic resistance and virulence determinants. Front. Microbiol..

[34] Shuchi S., Satija S., Jais M.B. (2025). *Elizabethkingia anophelis*: An uncommon cause of hospital-acquired septic meningitis and a rare cause of community-acquired meningitis in children: A case series. Asian J. Med. Sci..

[35] Janda J.M., Lopez D.L. (2017). Mini review: New pathogen profiles: *Elizabethkingia anophelis*. Diagn. Microbiol. Infect. Dis..

[36] Wang J., Su J., Li N., Yang Y., Li J., Jin J. (2026). Clinical Characteristics and Drug Resistance Analysis of *Elizabethkingia meningoseptica* Colonization/Infection in Hospital Settings. J. Hosp. Infect..

[37] Chen M., Cai Y., Shi Q., Xu A., Tang T., Qian J., Yu S., Zhu H., Xu J., Li J. (2026). Antimicrobial management and infection outcomes of *Elizabethkingia* spp. co-detection in lower respiratory tract: A real-world mNGS-based observational study. Int. J. Infect. Dis..

[38] González L.J., Vila A.J. (2012). Carbapenem resistance in *Elizabethkingia meningoseptica* is mediated by metallo-β-lactamase BlaB. Antimicrob. Agents Chemother..

[39] Wu C., Xiong L., Liao Q., Zhang W., Xiao Y., Xie Y. (2024). Clinical manifestations, antimicrobial resistance and genomic feature analysis of multidrug-resistant *Elizabethkingia* strains. Ann. Clin. Microbiol. Antimicrob..

[40] Sahu R., Pathi B.K., Panigrahi K., Mohapatra S., Sahoo J.P. (2025). A Retrospective Study on the Antimicrobial Susceptibility Patterns of *Elizabethkingia meningoseptica* Specimens Among Adult Patients. Cureus.

[41] Gawande R., Bamnote P., Bankar N., Peshattiwar P. (2026). *Elizabethkingia meningoseptica*: An emerging nosocomial pathogen. Int. J. Health Allied Sci..

[42] Zhang Q., Cong S., Wang Y. (2026). From cognition to response: Clinical challenges, antibiotic resistance mechanisms, and prevention and control strategies of *Elizabethkingia meningoseptica* infections. Front. Microbiol..

[43] Chen S., Soehnlen M., Blom J., Terrapon N., Henrissat B., Walker E.D. (2019). Comparative genomic analyses reveal diverse virulence factors and antimicrobial resistance mechanisms in clinical *Elizabethkingia meningoseptica* strains. PLoS ONE.

[44] Tsai Y.-M., Liu C.-M., Chen H.-C., Yang T.-H., Huang P.-S., Hsu Y.-L., Baranwal M., Chiang M.-H. (2025). Imipenem-induced outer membrane vesicles from *Elizabethkingia anophelis* inhibit biofilm formation and shift nosocomial pathogen dynamics. Int. J. Med. Microbiol..

[45] Hammers D., Carothers K., Lee S. (2022). The Role of Bacterial Proteases in Microbe and Host-microbe Interactions. Curr. Drug Targets.

[46] Frees D., Brøndsted L., Ingmer H. (2013). Bacterial proteases and virulence. Subcell. Biochem..

[47] Mwanza E.P., Hugo A., Charimba G., Hugo C.J. (2022). Pathogenic Potential and Control of *Chryseobacterium* Species from Clinical, Fish, Food and Environmental Sources. Microorganisms.

[48] Eggert T., Brockmeier U., Dröge M.J., Quax W.J., Jaeger K.-E. (2003). Extracellular lipases from *Bacillus subtilis*: Regulation of gene expression and enzyme activity by amino acid supply and external pH. FEMS Microbiol. Lett..

[49] Urban A., Leipelt M., Eggert T., Jaeger K.E. (2001). DsbA and DsbC affect extracellular enzyme formation in *Pseudomonas aeruginosa*. J. Bacteriol..

[50] Kouker G., Jaeger K.E. (1987). Specific and sensitive plate assay for bacterial lipases. Appl. Environ. Microbiol..

[51] Chen S., Soehnlen M., Downes F.P., Walker E.D. (2017). Insights from the draft genome into the pathogenicity of a clinical isolate of *Elizabethkingia meningoseptica* Em3. Stand. Genom. Sci..

[52] Ciofu O., Moser C., Jensen P.Ø., Høiby N. (2022). Tolerance and resistance of microbial biofilms. Nat. Rev. Microbiol..

[53] Donlan Rodney M., Costerton J.W. (2002). Biofilms: Survival Mechanisms of Clinically Relevant Microorganisms. Clin. Microbiol. Rev..

[54] Hu S., Lv Y., Xu H., Zheng B., Xiao Y. (2022). Biofilm formation and antibiotic sensitivity in *Elizabethkingia anophelis*. Front. Cell. Infect. Microbiol..

[55] Yang C.H., Su P.W., Moi S.H., Chuang L.Y. (2019). Biofilm Formation in *Acinetobacter Baumannii*: Genotype-Phenotype Correlation. Molecules.

[56] Shenkutie A.M., Yao M.Z., Siu G.K., Wong B.K.C., Leung P.H. (2020). Biofilm-Induced Antibiotic Resistance in Clinical *Acinetobacter baumannii* Isolates. Antibiotics.

[57] Hashemzadeh M., Dezfuli A.A.Z., Nashibi R., Jahangirimehr F., Akbarian Z.A. (2021). Study of biofilm formation, structure and antibiotic resistance in *Staphylococcus saprophyticus* strains causing urinary tract infection in women in Ahvaz, Iran. New Microbes New Infect..

[58] Fauzia K.A., Miftahussurur M., Syam A.F., Waskito L.A., Doohan D., Rezkitha Y.A.A., Matsumoto T., Tuan V.P., Akada J., Yonezawa H. (2020). Biofilm Formation and Antibiotic Resistance Phenotype of *Helicobacter pylori* Clinical Isolates. Toxins.

[59] Shadkam S., Goli H.R., Mirzaei B., Gholami M., Ahanjan M. (2021). Correlation between antimicrobial resistance and biofilm formation capability among *Klebsiella pneumoniae* strains isolated from hospitalized patients in Iran. Ann. Clin. Microbiol. Antimicrob..

[60] Liu H.Y., Prentice E.L., Webber M.A. (2024). Mechanisms of antimicrobial resistance in biofilms. npj Antimicrob. Resist..

[61] Irizarry-Tardi N., Karunathilaka J.C., Gallina N.L.F., Krishnakumar A., Wang W., Rahimi R., Bhunia A.K. (2025). Regulation and Therapeutic Intervention of Bacterial Biofilms. Curr. Clin. Microbiol. Rep..

[62] Lebeaux D., Ghigo J.-M., Beloin C. (2014). Biofilm-Related Infections: Bridging the Gap between Clinical Management and Fundamental Aspects of Recalcitrance toward Antibiotics. Microbiol. Mol. Biol. Rev..

[63] Chandran V.T., Thangaraj M., Gnanakumar T., Suriakumar J. (2025). Advancements in Targeting Biofilm-Associated Infections: Novel Therapeutic Approaches and Challenges. South East. Eur. J. Public Health.

[64] Chaiwarit T., Duangsonk K., Yuantrakul S., Chanabodeechalermrung B., Khangtragool W., Brachais C.-H., Chambin O., Jantrawut P. (2024). Synthesis of Carboxylate-Dialdehyde Cellulose to Use as a Component in Composite Thin Films for an Antibacterial Material in Wound Dressing. ACS Omega.

[65] Fawaz S., Barton S., Whitney L., Swinden J., Nabhani-Gebara S. (2019). Stability of Meropenem After Reconstitution for Administration by Prolonged Infusion. Hosp. Pharm..

[66] Zhou J., Qian Y., Lang Y., Zhang Y., Tao X., Moya B., Sayed A.R.M., Landersdorfer C.B., Shin E., Werkman C. (2024). Comprehensive stability analysis of 13 β-lactams and β-lactamase inhibitors in in vitro media, and novel supplement dosing strategy to mitigate thermal drug degradation. Antimicrob. Agents Chemother..

[67] Tian H., Li W., Chen C., Yu H., Yuan H. (2023). Antibacterial Activity and Mechanism of Oxidized Bacterial Nanocellulose with Different Carboxyl Content. Macromol. Biosci..

[68] Luo H., Lan H., Cha R., Yu X., Gao P., Zhang P., Zhang C., Han L., Jiang X. (2021). Dialdehyde Nanocrystalline Cellulose as Antibiotic Substitutes against Multidrug-Resistant Bacteria. ACS Appl. Mater. Interfaces.

[69] Kokol V., Novak S., Kononenko V., Kos M., Vivod V., Gunde-Cimerman N., Drobne D. (2023). Antibacterial and degradation properties of dialdehyded and aminohexamethylated nanocelluloses. Carbohydr. Polym..

[70] Amador C.I., Stannius R.O., Røder H.L., Burmølle M. (2021). High-throughput screening alternative to crystal violet biofilm assay combining fluorescence quantification and imaging. J. Microbiol. Methods.

[71] Ming D., Wang D., Cao F., Xiang H., Mu D., Cao J., Li B., Zhong L., Dong X., Zhong X. (2017). Kaempferol Inhibits the Primary Attachment Phase of Biofilm Formation in Staphylococcus aureus. Front. Microbiol..

[72] Mahanta U., Khandelwal M., Deshpande A.S. (2021). Antimicrobial surfaces: A review of synthetic approaches, applicability and outlook. J. Mater. Sci..

[73] Grari O., Ezrari S., El Yandouzi I., Benaissa E., Ben Lahlou Y., Lahmer M., Saddari A., Elouennass M., Maleb A. (2025). A comprehensive review on biofilm-associated infections: Mechanisms, diagnostic challenges, and innovative therapeutic strategies. Microbe.

[74] Zou Y., Qu Y., Zhang Y., Yu Q. (2025). Multifunctional antibiofilm materials: Surface engineering and nanotherapeutic strategies for biofilm prevention and eradication. Acta Biomater..

[75] Zhang X., Zheng Z., Zhang Y., Wang M., Lu R. (2026). The clinical challenge of *Elizabethkingia* infections: Multidrug resistance, epidemiology, and management strategies. Microb. Pathog..

[76] Li H., Fan D., Liu X., Chen Y., Xiao J., Hou C., Zeng W., Chen F. (2026). Stage-specific therapeutic strategies for combating bacterial biofilm infections: Recent advances and future perspectives. J. Control. Release.

[77] Bauer A.W., Kirby W.M., Sherris J.C., Turck M. (1966). Antibiotic susceptibility testing by a standardized single disk method. Am. J. Clin. Pathol..

[78] Schröttner P., Gunzer F., Schüppel J., Rudolph W. (2016). Identification of rare bacterial pathogens by 16S rRNA gene sequencing and MALDI-TOF MS. J. Vis. Exp..

[79] Cobo F., Pérez-Carrasco V., Martín-Hita L., García-Salcedo J.A., Navarro-Marí J.M. (2023). Comparative evaluation of MALDI-TOF MS and 16S rRNA gene sequencing for the identification of clinically relevant anaerobic bacteria: Critical evaluation of discrepant results. Anaerobe.

[80] Buxton R. (2005). Blood agar plates and hemolysis protocols. Am. Soc. Microbiol..

[81] Edberg S.C., Gallo P., Kontnick C. (1996). Analysis of the Virulence Characteristics of Bacteria Isolated from Bottled, Water Cooler, and Tap Water. Microb. Ecol. Health Dis..

[82] Puah S.M., Fong S.P., Kee B.P., Puthucheary S.D., Chua K.H. (2022). Molecular identification and biofilm-forming ability of *Elizabethkingia* species. Microb. Pathog..

[83] Clinical and Laboratory Standards Institute (2016). Performance Standards for Antimicrobial Susceptibility Testing.

[84] Magiorakos A.P., Srinivasan A., Carey R.B., Carmeli Y., Falagas M.E., Giske C.G., Harbarth S., Hindler J.F., Kahlmeter G., Olsson-Liljequist B. (2012). Multidrug-resistant, extensively drug-resistant and pandrug-resistant bacteria: An international expert proposal for interim standard definitions for acquired resistance. Clin. Microbiol. Infect..

[85] Chaiwarit T., Panyathip R., Yuantrakul S., Duangsonk K., Panraksa P., Rachtanapun P., Jantanasakulwong K., Jantrawut P. (2025). Synthesis and Fabrication of Dialdehyde Cellulose/PVA Films Incorporating Carbon Quantum Dots for Active Packaging Applications. Polymers.

[86] Abral H., Pratama A.B., Handayani D., Mahardika M., Aminah I., Sandrawati N., Sugiarti E., Muslimin A.N., Sapuan S.M., Ilyas R.A. (2021). Antimicrobial Edible Film Prepared from Bacterial Cellulose Nanofibers/Starch/Chitosan for a Food Packaging Alternative. Int. J. Polym. Sci..

