# Supplementary material for: Comprehensive Phenotypic Characterization of Clinical Elizabethkingia Isolates and Evaluation of the Antimicrobial and Anti-Biofilm Activity of Dialdehyde Cellulose
Source: Int J Mol Sci. 2026 Jul 18;27(14):6392. doi: 10.3390/ijms27146392 (PMC13409810; doi:10.3390/ijms27146392)
Supplement: Supplementary file 1 [file ijms-27-06392-s001.zip › ijms-4430856-supplementary.pdf]

Supplementary Materials

Supplementary Table S1. Minimum inhibitory concentrations (MICs) and susceptibility profiles of *Elizabethkingia* isolates (n = 49).

Isolate	TZ	CZ	TX	FEP	CX	IP	ME	G	AN	CI	LX	ST	Category
**CMEA01**	≤8 (S)	>32 (R)	>32 (R)	32 (R)	>32 (R)	≥16 (R)	≥16 (R)	4 (S)	≤8 (S)	≥4 (R)	2 (S)	2 (S)	**MDR**
**CMEA02**	≤8 (S)	>32 (R)	32 (I)	16 (I)	>32 (R)	≥16 (R)	≥16 (R)	>8 (R)	32 (I)	0.5 (S)	0.25 (S)	≤1 (S)	**MDR**
**CMEA03**	≤8 (S)	>32 (R)	32 (I)	16 (I)	>32 (R)	≥16 (R)	≥16 (R)	>8 (R)	32 (I)	0.5 (S)	0.5 (S)	2 (S)	**MDR**
**CMEA04**	≤8 (S)	>32 (R)	>32 (R)	32 (R)	>32 (R)	≥16 (R)	≥16 (R)	≤2 (S)	≤8 (S)	1 (S)	2 (S)	2 (S)	**MDR**
**CMEA07**	≤8 (S)	>32 (R)	32 (I)	16 (I)	32 (I)	≥16 (R)	≥16 (R)	>8 (R)	≤8 (S)	1 (S)	0.5 (S)	2 (S)	**MDR**
**CMEA08**	≤8 (S)	>32 (R)	>32 (R)	16 (I)	>32 (R)	≥16 (R)	≥16 (R)	>8 (R)	16 (S)	0.5 (S)	0.25 (S)	2 (S)	**MDR**
**CMEA09**	≤8 (S)	>32 (R)	>32 (R)	32 (R)	>32 (R)	≥16 (R)	≥16 (R)	>8 (R)	≤8 (S)	1 (S)	1 (S)	≤1 (S)	**MDR**
**CMEA12**	≤8 (S)	>32 (R)	32 (I)	16 (I)	32 (I)	>16 (R)	>16 (R)	>8 (R)	32 (I)	2 (I)	1 (S)	2 (S)	**MDR**
**CMEA13**	32 (I)	4 (S)	>32 (R)	32 (R)	>32 (R)	4 (S)	4 (S)	>8 (R)	16 (S)	0.25 (S)	0.5 (S)	≤1 (S)	**MDR**
**CMEA14**	≤8 (S)	>32 (R)	32 (I)	16 (I)	16 (I)	>16 (R)	>16 (R)	>8 (R)	32 (I)	>2 (R)	2 (S)	2 (S)	**MDR**
**CMEA15**	≤6 (S)	>32 (R)	32 (I)	32 (R)	32 (I)	>16 (R)	>16 (R)	>8 (R)	32 (I)	1 (S)	1 (S)	2 (S)	**MDR**
**CMEA22**	≤8 (S)	>32 (R)	>32 (R)	>32 (R)	>32 (R)	>16 (R)	>16 (R)	>8 (R)	>32 (R)	>2 (R)	>8 (R)	>4 (R)	**XDR**
**CMEA26**	≤8 (S)	>32 (R)	32 (I)	16 (I)	32 (I)	16 (R)	>16 (R)	>8 (R)	16 (S)	1 (S)	1 (S)	2 (S)	**MDR**
**CMEA27**	≤8 (S)	>32 (R)	>32 (R)	32 (R)	>32 (R)	>16 (R)	>16 (R)	>8 (R)	32 (I)	2 (I)	2 (S)	2 (S)	**MDR**
**CMEM28**	≤8 (S)	>32 (R)	>32 (R)	32 (R)	>32 (R)	8 (I)	16 (R)	8 (I)	16 (S)	1 (S)	1 (S)	≤1 (S)	**MDR**
**CMEA29**	≤8 (S)	32 (R)	16 (I)	4 (S)	8 (S)	16 (R)	>16 (R)	≤2 (S)	≤8 (S)	0.5 (S)	1 (S)	≤1 (S)	**Susceptible**
**CMEA30**	≤8 (S)	>32 (R)	32 (I)	8 (S)	16 (I)	>16 (R)	>16 (R)	>8 (R)	32 (I)	2 (I)	1 (S)	4 (R)	**MDR**
**CMEA31**	≤8 (S)	>32 (R)	>32 (R)	16 (I)	>32 (R)	>16 (R)	>16 (R)	>8 (R)	≤8 (S)	1 (S)	1 (S)	≤1 (S)	**MDR**
**CMEA32**	≤8 (S)	>32 (R)	32 (I)	16 (I)	32 (I)	4 (S)	8 (I)	>8 (R)	16 (S)	>2 (R)	1 (S)	≤1 (S)	**MDR**
**CMEA33**	≤8 (S)	>32 (R)	>32 (R)	>32 (R)	>32 (R)	>16 (R)	>16 (R)	>8 (R)	>32 (R)	>2 (R)	>8 (R)	>4 (R)	**XDR**
**CMEA34**	≤8 (S)	>32 (R)	32 (I)	16 (I)	32 (I)	>16 (R)	>16 (R)	>8 (R)	32 (I)	>2 (R)	>8 (R)	≤1 (S)	**XDR**
**CMEA36**	≤8 (S)	>32 (R)	16 (I)	8 (S)	32 (I)	16 (R)	>16 (R)	>8 (R)	32 (I)	0.5 (S)	0.5 (S)	≤1 (S)	**MDR**
**CMEA37**	≤8 (S)	>32 (R)	32 (I)	8 (S)	16 (I)	4 (S)	8 (I)	>8 (R)	≤8 (S)	2 (I)	1 (S)	≤1 (S)	**MDR**
**CMEA39**	64 (I)	>32 (R)	>32 (R)	>32 (R)	>32 (R)	4 (S)	8 (I)	>8 (R)	16 (S)	>2 (R)	2 (S)	4 (R)	**MDR**
**CMEA40**	≤8 (S)	>32 (R)	>32 (R)	>32 (R)	>32 (R)	>16 (R)	>16 (R)	>8 (R)	>32 (R)	>2 (R)	>8 (R)	>4 (R)	**XDR**
**CMEA41**	>64 (R)	>32 (R)	>32 (R)	>32 (R)	>32 (R)	>16 (R)	>16 (R)	>8 (R)	16 (S)	>2 (R)	>8 (R)	>4 (R)	**XDR**

**CMEA42**	≤8 (S)	32 (R)	16 (I)	4 (S)	16 (I)	>16 (R)	>16 (R)	>8 (R)	32 (I)	>2 (R)	>8 (R)	≤1 (S)	**MDR**
**CMEM44**	≤8 (S)	>32 (R)	>32 (R)	32 (R)	>32 (R)	8 (I)	16 (R)	4 (S)	16 (S)	>2 (R)	2 (S)	≤1 (S)	**MDR**
**CMEA45**	>64 (R)	>32 (R)	>32 (R)	>32 (R)	>32 (R)	16 (R)	16 (R)	>8 (R)	>32 (R)	2 (I)	2 (S)	4 (R)	**XDR**
**CMEA48**	≤8 (S)	>32 (R)	32 (I)	16 (I)	>32 (R)	16 (R)	>16 (R)	>8 (R)	32 (I)	2 (I)	1 (S)	2 (S)	**MDR**
**CMEA50**	≤8 (S)	>32 (R)	32 (I)	16 (I)	32 (I)	>16 (R)	>16 (R)	>8 (R)	32 (I)	1 (S)	1 (S)	≤1 (S)	**MDR**
**CMEA51**	≤8 (S)	>32 (R)	32 (I)	16 (I)	32 (I)	>16 (R)	>16 (R)	>8 (R)	>32 (R)	1 (S)	1 (S)	≤1 (S)	**MDR**
**CMEA52**	≤8 (S)	>32 (R)	>32 (R)	16 (I)	32 (I)	>16 (R)	>16 (R)	>8 (R)	>32 (R)	2 (I)	1 (S)	2 (S)	**MDR**
**CMEA58**	≤8 (S)	>32 (R)	32 (I)	16 (I)	32 (I)	>8 (R)	>8 (R)	>8 (R)	>32 (R)	2 (I)	1 (S)	2 (S)	**MDR**
**CMEA60**	≤8 (S)	>32 (R)	>32 (R)	16 (I)	32 (I)	16 (R)	>16 (R)	>8 (R)	>32 (R)	2 (I)	1 (S)	2 (S)	**MDR**
**CMEA64**	≤8 (S)	>32 (R)	32 (I)	8 (S)	16 (I)	16 (R)	>16 (R)	>8 (R)	32 (I)	>2 (R)	2 (S)	≤1 (S)	**MDR**
**CMEA65**	16 (S)	>32 (R)	32 (I)	16 (I)	>32 (R)	16 (R)	>16 (R)	>8 (R)	32 (I)	>2 (R)	4 (I)	2 (S)	**XDR**
**CMEMi66**	≤8 (S)	>32 (R)	32 (I)	16 (I)	32 (I)	>16 (R)	>16 (R)	≤2 (S)	≤8 (S)	1 (S)	1 (S)	≤1 (S)	**Susceptible**
**CMEM67**	16 (S)	>32 (R)	>32 (R)	>32 (R)	>32 (R)	16 (R)	16 (R)	>8 (R)	>32 (R)	0.5 (S)	0.5 (S)	≤1 (S)	**MDR**
**CMEA71**	≤8 (S)	32 (R)	32 (I)	32 (R)	>32 (R)	>16 (R)	>16 (R)	>8 (R)	>32 (R)	2 (I)	1 (S)	2 (S)	**MDR**
**CMEA73**	≤8 (S)	>32 (R)	32 (I)	16 (I)	16 (I)	16 (R)	16 (R)	≤2 (S)	≤8 (S)	2 (I)	1 (S)	≤1 (S)	**MDR**
**CMEM76**	≤8 (S)	>32 (R)	>32 (R)	>32 (R)	>32 (R)	16 (R)	16 (R)	>8 (R)	32 (I)	1 (S)	1 (S)	≤1 (S)	**MDR**
**CMEMi79**	≤8 (S)	>32 (R)	>32 (R)	32 (R)	>32 (R)	>16 (R)	>16 (R)	>8 (R)	32 (I)	1 (S)	0.5 (S)	≤1 (S)	**MDR**
**CMEM86**	≤8 (S)	>32 (R)	>32 (R)	>32 (R)	>32 (R)	16 (R)	16 (R)	>8 (R)	32 (I)	1 (S)	1 (S)	≤1 (S)	**MDR**
**CMEA89**	≤8 (S)	>32 (R)	16 (I)	8 (S)	32 (I)	16 (R)	>16 (R)	>8 (R)	32 (I)	2 (I)	2 (S)	≤1 (S)	**MDR**
**CMEA98**	≤8 (S)	>32 (R)	>32 (R)	>32 (R)	>32 (R)	>16 (R)	>16 (R)	>8 (R)	>32 (R)	>2 (R)	>8 (R)	>4 (R)	**XDR**
**CMEA110**	16 (S)	>32 (R)	>32 (R)	32 (R)	>32 (R)	16 (R)	>16 (R)	>8 (R)	>32 (R)	1 (S)	0.5 (S)	>4 (R)	**XDR**
**CMEM111**	≤8 (S)	>32 (R)	>32 (R)	>32 (R)	>32 (R)	8 (I)	8 (I)	>8 (R)	32 (I)	0.5 (S)	0.5 (S)	≤1 (S)	**MDR**
**CMEA112**	≤8 (S)	>32 (R)	32 (I)	16 (I)	16 (I)	16 (R)	>16 (R)	>8 (R)	>32 (R)	1 (S)	0.5 (S)	≤1 (S)	**MDR**

MIC values (mg/L) for each antimicrobial agent are presented with the corresponding categorical interpretation in parentheses: S, susceptible; I, intermediate; R, resistant. Antimicrobials tested were: TZ, piperacillin/tazobactam; CZ, ceftazidime; TX, ceftriaxone; FEP, cefepime; CX, cefotaxime; IP, imipenem; ME, meropenem; G, gentamicin; AN, amikacin; CI, ciprofloxacin; LX, levofloxacin; ST, trimethoprim/sulfamethoxazole. Isolate codes are shown in the first column. Multidrug-resistant (MDR), Pan drug-resistant (PDR) and extensively drug-resistant (XDR) phenotypes were defined according to international consensus criteria.

Supplementary Table S2. Virulence enzyme production, hemolysis patterns and biofilm formation of selected *Elizabethkingia* isolates.

Strain	Protease			Lipase			Lecithinase			Hemolysis			Biofilm Formation
	24Hr	48Hr	72Hr	24Hr	48Hr	72Hr	24Hr	48Hr	72Hr	24Hr	48Hr	72Hr	
**CMEA1**	+	+	+	-	-	-	-	-	-	Alpha	Alpha	Beta	Strong
**CMEA2**	+	+	+	-	-	-	-	-	-	None	Alpha	Beta	None
**CMEA3**	+	+	+	-	-	-	-	-	-	Alpha	Alpha	Beta	None
**CMEA4**	+	+	+	-	-	-	-	-	-	Alpha	Alpha	Beta	None
**CMEA7**	+	+	+	-	-	-	-	-	-	Alpha	Alpha	Beta	Weak
**CMEA8**	+	+	+	-	-	-	-	-	-	Alpha	Alpha	Beta	Strong
**CMEA9**	+	+	+	-	-	-	-	-	-	Alpha	Alpha	Beta	None
**CMEA12**	+	+	+	-	-	-	-	-	-	None	Alpha	Beta	None
**CMEA13**	+	+	+	-	-	-	-	-	-	Alpha	Alpha	Beta	None
**CMEA14**	+	+	+	-	-	-	-	-	-	Alpha	Alpha	Beta	None
**CMEA15**	+	+	+	-	-	-	-	-	-	Alpha	Alpha	Beta	Weak
**CMEA22**	+	+	+	-	-	-	-	-	-	Alpha	Alpha	Beta	Moderate
**CMEA26**	+	+	+	-	-	-	-	-	-	Alpha	Alpha	Beta	Weak
**CMEA27**	+	+	+	-	-	-	-	-	-	Alpha	Alpha	Beta	Moderate
**CMEM28**	+	+	+	-	-	-	-	-	-	None	Alpha	Beta	Strong
**CMEA29**	+	+	+	-	-	-	-	-	-	Alpha	Alpha	Beta	Moderate
**CMEA30**	+	+	+	-	-	-	-	-	-	Alpha	Alpha	Beta	None
**CMEA31**	+	+	+	-	-	-	-	-	-	Alpha	Alpha	Beta	None
**CMEA32**	+	+	+	-	-	-	-	-	-	Alpha	Alpha	Beta	Strong
**CMEA33**	-	+	+	-	-	-	-	-	-	None	Alpha	Beta	None
**CMEA34**	-	+	+	-	-	-	-	-	-	None	Alpha	Beta	None
**CMEA36**	+	+	+	-	-	-	-	-	-	Alpha	Alpha	Beta	Weak
**CMEA37**	+	+	+	-	-	-	-	-	-	Alpha	Beta	Beta	Strong
**CMEA39**	+	+	+	-	-	-	-	-	-	Alpha	Alpha	Beta	Strong
**CMEA40**	+	+	+	-	-	-	-	-	-	Alpha	Alpha	Beta	Strong
**CMEA41**	+	+	+	-	-	-	-	-	-	Alpha	Alpha	Beta	Weak
**CMEA42**	+	+	+	-	-	-	-	-	-	Alpha	Alpha	Beta	Moderate
**CMEM44**	-	+	+	-	-	-	-	-	-	Alpha	Alpha	Beta	Strong
**CMEA45**	+	+	+	-	-	-	-	-	-	Alpha	Alpha	Beta	Strong
**CMEA48**	-	+	+	-	-	-	-	-	-	Alpha	Alpha	Beta	Weak
**CMEA50**	+	+	+	-	-	-	-	-	-	Alpha	Alpha	Beta	Moderate
**CMEA51**	+	+	+	-	-	-	-	-	-	Alpha	Alpha	Beta	None
**CMEA52**	+	+	+	-	-	-	-	-	-	Alpha	Alpha	Beta	None
**CMEA58**	+	+	+	-	-	-	-	-	-	Alpha	Alpha	Beta	Strong
**CMEA60**	+	+	+	-	-	-	-	-	-	Alpha	Alpha	Beta	Strong
**CMEA64**	+	+	+	-	-	-	-	-	-	Alpha	Alpha	Beta	Strong
**CMEA65**	+	+	+	-	-	-	-	-	-	Alpha	Alpha	Beta	Strong
**CMEMi66**	+	+	+	-	-	-	-	-	-	Alpha	Beta	Beta	None
**CMEM67**	+	+	+	-	-	-	-	-	-	Alpha	Alpha	Beta	Strong
**CMEA71**	+	+	+	-	-	-	-	-	-	Alpha	Alpha	Beta	None
**CMEA73**	+	+	+	-	-	-	-	-	-	Alpha	Alpha	Beta	None

**CMEM76**	+	+	+	-	-	-	-	-	-	Alpha	Alpha	Beta	Strong
**CMEMi79**	+	+	+	-	-	-	-	-	-	Alpha	Alpha	Alpha	None
**CMEM86**	+	+	+	-	-	-	-	-	-	Alpha	Alpha	Beta	Strong
**CMEA89**	-	+	+	-	-	-	-	-	-	Alpha	Alpha	Alpha	None
**CMEA98**	+	+	+	-	-	-	-	-	-	Alpha	Alpha	Alpha	Strong
**CMEA110**	+	+	+	-	-	-	-	-	-	Alpha	Alpha	Beta	None
**CMEM111**	-	+	+	-	-	-	-	-	-	Alpha	Alpha	Alpha	None
**CMEA112**	+	+	+	-	-	-	-	-	-	Alpha	Beta	Beta	Strong

A total of 49 bacterial isolates were screened for the production of virulence factors, including protease, lipase, lecithinase, and hemolysin. Protease, lipase, and lecithinase activities were recorded at 24, 48, and 72 hours of incubation and are indicated as positive (+) or negative (-). Hemolysis patterns were classified as alpha (partial), beta (complete), or none after 24,48,72 hours of incubation on blood agar. Biofilm formation phenotypes are classified as none, weak, moderate and strong respectively. Representative data for selected isolates are shown.

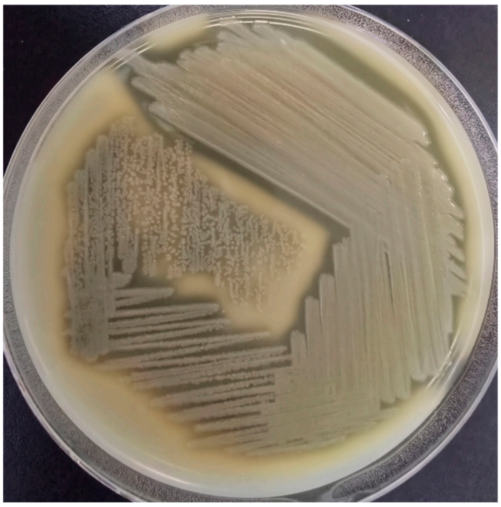
(A)

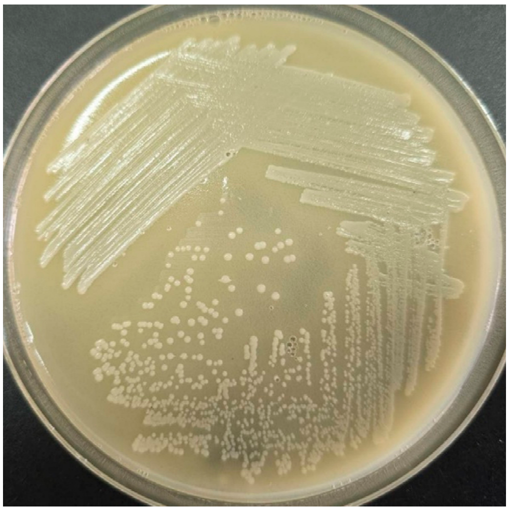
(B)

Supplementary Figure S1. Representative assessment of virulence-associated extracellular enzyme activities in a representative *Elizabethkingia anophelis* isolate after 24 h of incubation at 37 °C. (A) Positive protease activity on skimmed milk agar, demonstrated by a distinct zone of casein hydrolysis surrounding the bacterial growth. **(B)** No evidence of lipase or lecithinase activity on egg yolk agar. Neither an iridescent sheen, indicative of lipase activity, nor a white opaque precipitate surrounding the colonies, indicative of lecithinase activity, was observed after 48 h of incubation.

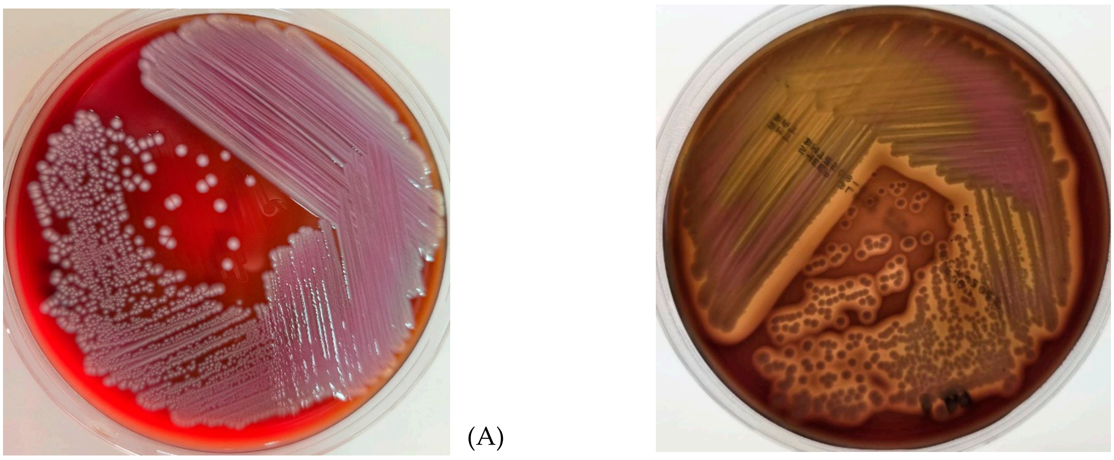

(A) (B)

Supplementary Figure S2. Representative hemolytic activity of *Elizabethkingia* spp. after 48 h of incubation at 37 °C on 5% sheep blood agar. (A) Representative *Elizabethkingia anophelis* isolate exhibiting α-hemolysis, characterized by partial hemolysis with a greenish discoloration surrounding the bacterial colonies. **(B)** Representative *Elizabethkingia miricola* isolate exhibiting β-hemolysis, characterized by complete hemolysis with a distinct clear zone surrounding the bacterial colonies.

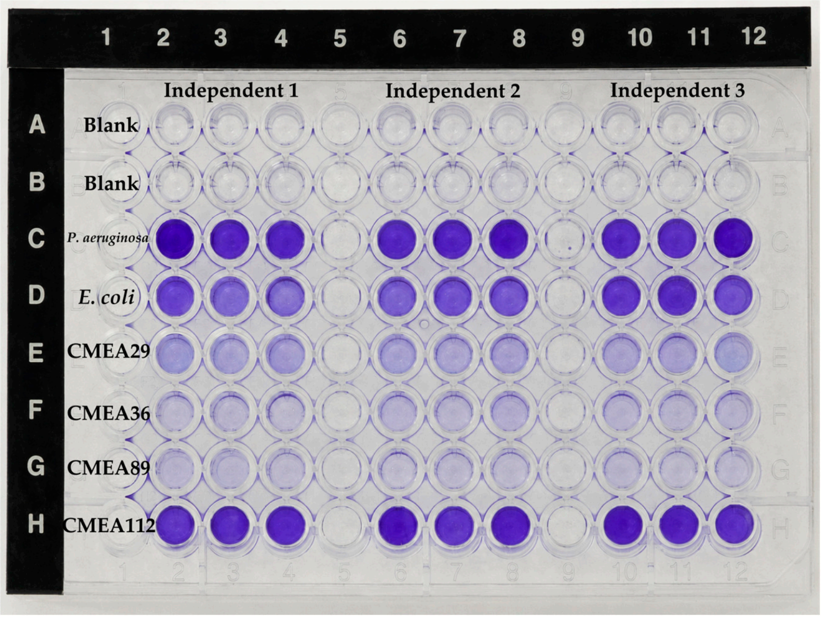

Supplementary Figure S3. Representative crystal violet biofilm formation assay of four *Elizabethkingia anophelis* isolates and two reference strains after 24 h of incubation at 37 °C. Biofilm formation was evaluated in a 96-well microtiter plate using the crystal violet staining assay, in which the intensity of purple staining reflects the amount of adherent biofilm biomass. Blank wells containing uninoculated medium served as negative controls. The figure shows three independent experiments, each performed in triplicate, with columns 2–4, 6–8, and 10–12 representing Independent Experiments 1, 2, and 3, respectively. *Pseudomonas aeruginosa* ATCC 27853 and *Escherichia coli* ATCC 25922 served as reference strains and both exhibited strong biofilm formation. Among the *E. anophelis* isolates, CMEA29 was classified as a moderate biofilm producer, CMEA36 as a weak biofilm producer, CMEA89 as a non-biofilm producer, and CMEA112 as a strong biofilm producer.

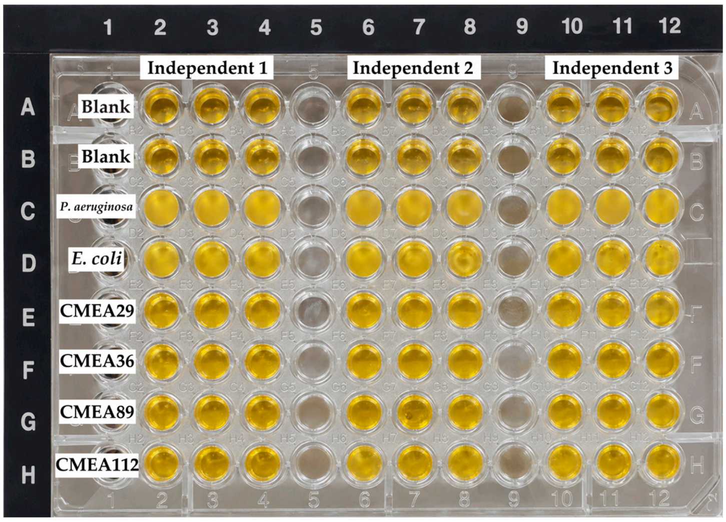

Supplementary Figure S4. Biofilm formation assessment of *Elizabethkingia anophelis* clinical isolates. Biofilm production inhibition assay in a DAC-coated 96-well plate. Bacterial cultures were incubated at 37°C for 24 hours. Rows A and B (blank negative controls) show no opacity, confirming sterility. Detectable growth, evidenced by increased opacity, was observed for *P. aeruginosa* (Row C) and *E. coli* (Row D) compared to the four *E. anophelis* isolates (CMEA29, CMEA36, CMEA89, and CMEA112; Rows E–H, respectively).

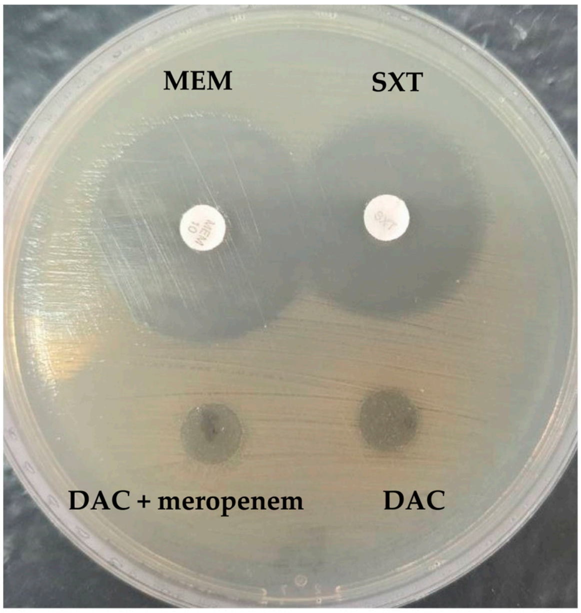

A.

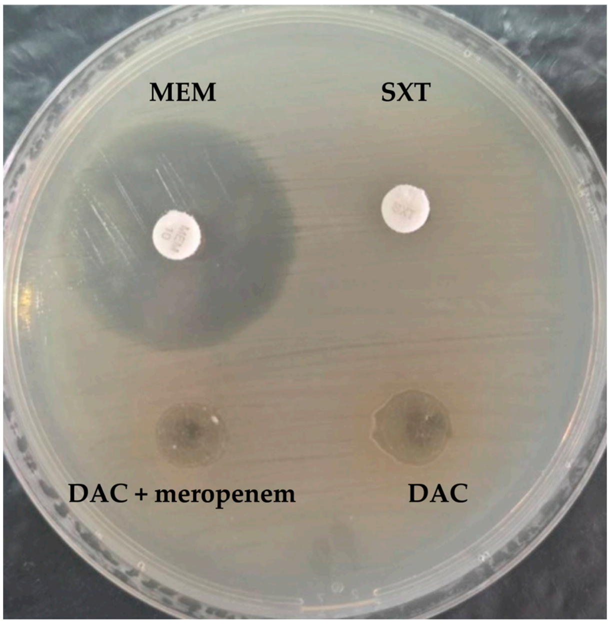

B.

Supplementary Figure S5. Phenotypic validation of antimicrobial activity using reference control strains. Representative agar disk diffusion assay against **(A)** *Escherichia coli* ATCC 25922 and **(B)** *Pseudomonas aeruginosa* ATCC 27853. The assay included dialdehyde cellulose (DAC) film, meropenem-loaded DAC film (DAC + meropenem), and the control antibiotic disks meropenem (MEM) and trimethoprim–sulfamethoxazole (SXT). Representative images from three independent experiments are shown.

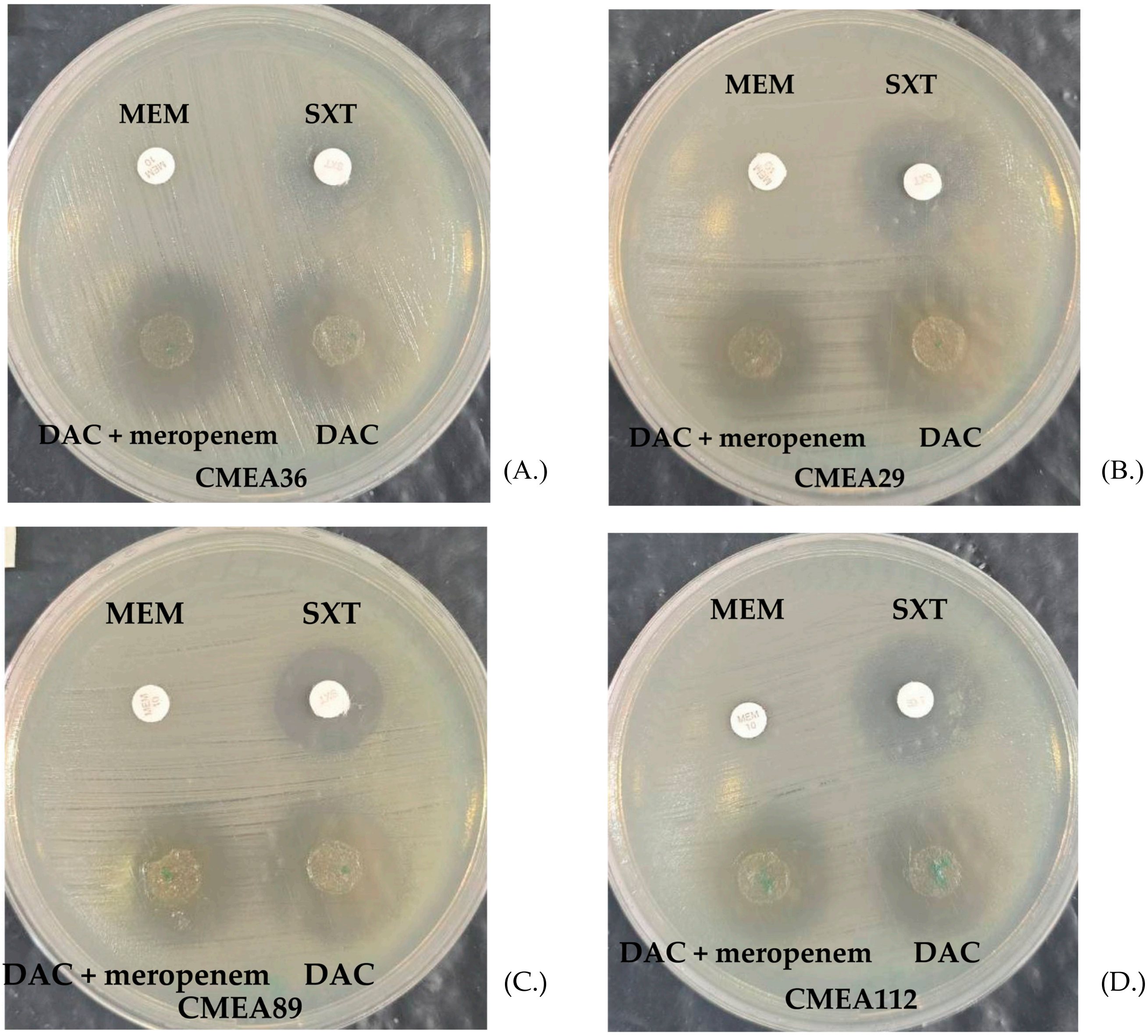

Supplementary Figure S6. Antimicrobial efficacy of dialdehyde cellulose (DAC) against four clinical *Elizabethkingia anophelis* isolates. Representative agar disk diffusion assay demonstrating the antimicrobial activity of dialdehyde cellulose (DAC) film, meropenem-loaded DAC film (DAC +meropenem), and control antibiotic disks against **(A)** CMEA29, **(B)** CMEA36, **(C)** CMEA89, and **(D)** CMEA112. Meropenem (MEM) and trimethoprim–sulfamethoxazole (SXT) disks were included as control antibiotic disks. Representative images from three independent experiments are shown.

Supplementary Table S3. GenBank accession numbers of the 16S rRNA gene sequences included in this study.

Strain	Acc. No.	Strain	Acc. No.
CMEA01	PZ669165	CMEA41	PZ670414
CMEA02	PZ669345	CMEA42	PZ672134
CMEA03	PZ669347	CMEM44	PZ670415
CMEA04	PZ672117	CMEA48	PZ672132
CMEA07	PZ672116	CMEA49	PZ672137
CMEA08	PZ672119	CMEA50	PZ670545
CMEA09	PZ669434	CMEA51	PZ672137
CMEA12	PZ669464	CMEA52	PZ672136
CMEA13	PZ669433	CMEA58	PZ672139
CMEA14	PZ672118	CMEA60	PZ672143
CMEA15	PZ672120	CMEA64	PZ672144
CMEA22	PZ672121	CMEA65	PZ672142
CMEA26	PZ672122	CMEMi66	PZ670559
CMEA27	PZ672123	CMEM67	PZ670560
CMEM28	PZ670046	CMEA71	PZ672108
CMEA29	PZ672127	CMEA73	PZ672109
CMEA30	PZ672126	CMEM76	PZ672110
CMEA31	PZ672128	CMEMi79	PZ670724
CMEA32	PZ672124	CMEM86	PZ672111
CMEA33	PZ672130	CMEA89	PZ672112
CMEA34	PZ672129	CMEA98	PZ672113
CMEA36	PZ672125	CMEA110	PZ672115
CMEA37	PZ672131	CMEM111	PZ670723
CMEA39	PZ672133	CMEA112	PZ672114
CMEA40	PZ672133		
